# The role of chemokines in type 1 diabetes‐associated neuropathy

**DOI:** 10.1002/edm2.419

**Published:** 2023-04-06

**Authors:** Evangelia Baldimtsi, Nektaria Papadopoulou‐Marketou, Maria C. Jenmalm, Jeanette Wahlberg

**Affiliations:** ^1^ Department of Endocrinology in Linköping, and Department of Health, Medicine and Caring Sciences Linköping University Linköping Sweden; ^2^ National and Kapodistrian University of Athens University Research Institute of Maternal and Child Health and Precision Medicine and UNESCO Chair on Adolescent Health Care Athens Greece; ^3^ Division of Inflammation and Infection, Department of Biomedical and Clinical Sciences Linköping University Linköping Sweden; ^4^ Faculty of Medical Sciences Örebro University Örebro Sweden

**Keywords:** autoimmunity, chemokines, diabetic neuropathy, type 1 diabetes

## Abstract

**Introduction:**

To investigate whether circulating chemokines contribute to the development of diabetic peripheral neuropathy (DPN) in patients with type 1 diabetes (T1D).

**Methods:**

Fifty‐two patients with childhood‐onset T1D (mean age 28 ± 4 yrs.; diabetes duration 19.5 **±** 5.5 yrs.) and 19 control subjects (mean age 26.5 ± 4.5 yrs.) were included in a cross‐sectional analysis of this long‐term longitudinal cohort study. A subgroup of 24 patients was followed prospectively for a further 10 yrs. Plasma levels of Th1‐ (CXCL9, CXCL10 and CXCL11), Th2‐ (CCL17 and CCL22) and Th17‐associated (CXCL8 and CCL20) chemokines were assessed in all subjects. Additionally, the TID patients underwent clinical examination and electroneurography.

**Results:**

The frequency of neuropathy was 21% (11/52). Higher levels of CXCL9 levels were found in patients with DPN compared with control subjects (*p* = .019); by contrast, no difference between patients without DPN and control subjects was seen after adjustment for multiple comparisons. In patients with DPN, CXCL10 correlated negatively with suralis MCV and suralis SNAP (rho −0.966, *p* < .001 and rho −0.738, *p* < .001, respectively) and was positively correlated with the vibration perception threshold (rho 0.639, *p* = .034), while CXCL8 correlated negatively with the cold perception threshold (rho −0.645, *p* = .032). The frequency of neuropathy increased to 54% (13/24) in the subgroup of 23 TID patients, followed by an additional 10 yrs.

**Conclusions:**

Changes in Th1‐ and Th17‐associated chemokines were associated with impaired peripheral sensory nerve function and nerve conduction after long disease duration in childhood‐onset T1D.

## Graphical abstract

Higher levels of CXCL9 levels were found in patients with DPN compared with control subjects (*p* = .019). Impaired peripheral sensory nerve function correlated to CXCL8(Th17) and CXCL10(Th1). The vibration perception threshold correlated positively to CXCL10 (Th1) levels.

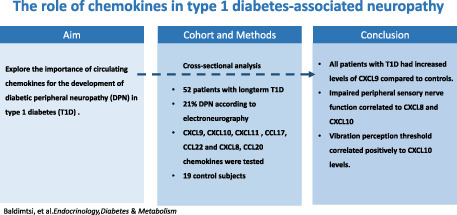

## 1 INTRODUCTION

Diabetic peripheral neuropathy (DPN) is a common microvascular complication in type 1 diabetes (T1D), and it occurs in nearly 50% after a long disease duration. Currently, much attention has been on the role of immunological factors such as chemokines in the development of diabetic neuropathy and neuropathic pain.^1^ Chemokines or chemotactic cytokines are small (8‐14 kDa) secreted peptides involved in innate and adaptive immune responses.^2^, ^3^, ^4^ Chemokines have a crucial role in establishing and maintaining the type of T helper preponderance in the immune response and may be used as reliably detectable markers for Th1‐, Th2‐ and Th17‐associated immunity.^3^, ^5^, ^6^, ^7^, ^8^, ^9^, ^10^ CXCL9, CXCL10 and CXCL11 are ligands for the receptor CXCR3 and are mainly secreted in response to interferon (IFN)‐ɣ, which TNF synergistically enhances. The CXCL9, CXCL10, CXCL11/CXCR3 axis regulates migration of activated Th1 cells, as they preferentially express CXCR3.^11^ On the contrary, the CCR4 ligands CCL17 (thymus‐ and activation‐regulated chemokine) and CCL22 (monocyte‐derived chemokine) are induced by the Th2 cytokines IL‐4 and IL‐13, and particularly recruit Th2 cells, preferentially expressing CCR4.^12^ The chemokines CXCL8 and CCL20 are induced by IL‐17 from Th17 cells, preferentially expressing CCR6, binding CCL20.^13^

Experimental evidence accumulated in the last years supports the concept that IFN‐ɣ inducible Th1‐associated chemokines CXCL9, CXCL10 and CXCL11 may play a critical role in developing several autoimmune disorders and in beta cell destruction in T1D.^3^, ^14^, ^15^ Elevated chemokines are involved in the development of diabetic complications such as retinopathy and nephropathy.^16^, ^17^ Furthermore, some studies indicate that CXCL9 is important in nociceptive transmission contributing to the development of diabetic pain, which is one of the most disabling symptoms in diabetic neuropathy.^18^, ^19^

Schwann cells, myelination cells of the peripheral nervous system, play an essential role in the pathogenesis and development of DPN. Studies indicate that the pathophysiological mechanism behind DPN is when the high glucose levels promote the expression of CXCR3 on CD8+ T cells and, on the contrary, induce the expression of CXCL9, CXCL10 and CXCL11 in Schwann cells, leading to the recruitment and infiltration of CD8^+^ T cells into diabetic peripheral tissues and increased cytotoxicity towards Schwann cells.^20^ In addition, Th17 cells may contribute to damage to peripheral neurons through the cytokine IL‐17, which may lead to diabetic neuropathy.^13^ However, while IL‐17 directly modulates neuronal cell behaviours in vitro, it is unclear if these effects also extend to T1D in vivo.^21^

Given these considerations, we hypothesized that diabetic neuropathy in individuals with childhood‐onset T1D is associated with increased levels of Th1 and Th17 chemokines and conversely with low levels of Th2 chemokines compared with control subjects and patients without neuropathy.

## 2 PATIENTS AND METHODS

### 2.1 Subjects and study design

Patients with childhood‐onset T1D (N = 102) prospectively followed in a longitudinal cohort study, since childhood and examined on at least one previous occasion with nerve conduction tests,^22^, ^23^ were asked to participate in the current cross‐sectional analysis at the exact center, the Endocrinology Department of Linköping University Hospital. In all, 52 patients were admitted to participate in the present study from 2007 to 2009. The diagnosis of type 1 diabetes was based on the presence of high blood glucose levels, increased glycated haemoglobin (HbA1c) in addition to the positive titre of at least one of the autoantibodies related to T1D; GAD (glutamic acid decarboxylase antibody) and IA‐2 (protein tyrosine phosphatase/insulinoma associated autoantibodies). All patients had been receiving intensive therapy from disease onset, which involved the administration of insulin by multiple injections daily or via an external subcutaneous infusion pump. Exclusion criteria were other causes of neuropathy, such as alcohol abuse or medication that might interfere with the neuropathy examination results or symptoms, such as epilepsy medication. At the time of the evaluation, no participant had a history of neurological or metabolic disease, alcohol abuse or medications known to have an adverse effect on peripheral nerve function.

For comparisons of chemokine levels, a control group of 19 healthy blood donors (eight men, 11 women; mean age 26.5 ± 4.5 yrs.) from Linköping, Sweden, were included. The control group did not perform the neurophysiological tests.

A follow‐up was planned with a third neurophysiological test after another decade. To examine if chemokine expression can predict the development of DPN, we intended to compare the two groups with and without DPN at the second follow‐up about chemokine expression a decade earlier. We used the chemokine levels obtained from the 19 healthy control subjects a decade earlier for comparisons. Due to the Covid‐19 pandemic, only half of the patients could be recruited. In total, a subgroup of 24 patients from the cohort of 52 patients was re‐examined with neurophysiological tests one more time from 2018 to 2021.

### 2.2 Neurophysiological examination and Quantitative Sensory tests

Confirmed diabetic peripheral neuropathy (DPN) was diagnosed by abnormalities in nerve conduction studies (Toronto Diabetic Neuropathy Expert Group).^15^ The neurophysiological examination (NSC) included bilateral measurements of both peroneal and median motor nerve conduction velocity (MCV), compound muscle action potential (CMAP) amplitude, sensory nerve action potential (SNAP), sural and median sensory nerve conduction velocity (SCV). In addition, all amplitudes (i.e. CMAPs, SNAPs) were measured from peak to peak, and sensory nerves were studied with an orthodromic recording of SNAPs. The results of the examination regarding neurophysiological tests have been published previously.^24^

QST was carried out bilaterally according to standardized procedures. A vibrating probe (Vibrameter; Somedic, Stockholm, Sweden, or Medoc Advanced Medical Systems, Ramat Yishai, Israel) was applied over the first metatarsal and the tibia (approximately 10 cm below the knee) for evaluations of the vibration perception thresholds (VPTs). Heat and cold temperature thresholds were determined using the Marstock technique with a temperature‐regulated probe (Thermotest; Somedic or Medoc Advanced Medical Systems) starting at 32°C and automatically changed by a rate of 1°C/s. The probe was applied over the dorsum of the feet and the tibial area. All measurements of sensation were estimated three times.

A staged approach determined the presence of diabetic neuropathy according to established criteria (i.e. stage 0 = no nerve conduction abnormality; 1a = nerve conduction abnormality only; 1b = nerve conduction abnormality + signs; 2a = nerve conduction + signs + symptoms; and 2b = nerve conduction abnormality + symptoms + more severe signs [i.e. 50% weakness of ankle dorsiflexion]).

Nerve conduction abnormality was defined as more than one abnormal attribute in two separate nerves. An abnormal point regarding nerve conduction velocity (NCV) was defined as < −2.33 SDS (first percentile or less) for the peroneal nerve (MCV and CMAP) and for the sural nerve (SCV and SNAP). Subclinical neuropathy is an electrophysiological abnormality of nerve function without clinical symptoms or signs. On the contrary, clinical neuropathy was defined as an electrophysiological abnormality of nerve function with clinical symptoms or signs.

### 2.3 Chemokine analyses

Chemokine concentrations used as markers for Th1‐, Th2‐ and Th17‐associated immunity (Table 2) were measured in EDTA plasma by Luminex multiple bead technologies (Milliplex Human Cytokine/Chemokine Kit, Millipore Corporation), according to the instructions provided by the manufacturer. Samples from patients and controls were analysed in parallel using the same reagent batch. The lower detection limits for chemokines in plasma were CXCL8, 1.6 pg/mL, CXCL9, 3.9 pg/mL, CXCL10 16 pg/mL, CXCL11 7.8 pg/mL, CCL17 3.9 pg/mL, CCL20 4.9 pg/mL and CCL22 80 pg/mL. Values below the detection limit were given half the value of the detection limit. All samples were analysed in duplicate, and the samples were reanalysed if the coefficient of variance (CV) was >15%.

### 2.4 Statistical analysis

Categorical variables are expressed as counts and percentages, or vice versa, continuous variables as mean (SD) or median [IQR]. The Shapiro–Wilk test was used to assess normality. The χ2‐test was used to explore the association between categorical variables, whereas independent samples *t*‐test or the Mann–Whitney *U* test were used to examine differences in demographic and clinical data between patients with and without DPN. Comparisons within the group were made with the Wilcoxon rank‐sum test. Independent samples Kruskal–Wallis test was used to examine the differences of continuous variables between controls and patients with and without DPN, while the Bonferroni correction for multiple comparisons was used for adjustment of significant values. Correlations (Spearman, Rho) were FDR‐adjusted based on the Benjamini–Hochberg method. *p*‐values <.05 were considered statistically significant. Statistical calculations were made using SPSS version 26.0 (IBM Corp, Armonk, NY, USA).

## 3 RESULTS

### 3.1 Demographic and clinical characteristics (Table 1 and Table 2)

**TABLE 1:** Demographics and clinical data for the two groups with type 1 diabetes without neuropathy (TID) and with neuropathy (TID + DPN).

	T1D *n* = 41	T1D + DPN *n* = 11	*p*‐value
Male	26 (50%)	6 (11%)	
Female	15 (29%)	5 (10%)	
Diabetes duration (years)	18.7 (± 5.6)	22.6 (± 3.6)	.011*
Mean age	26.8 (± 3.9)	30.7 (± 2.7)	ns
HbA1c (DCCT%)	7.4 (± 1.5)	7.7 (± 1.2)	ns
Fasting glucose (mmol/L)	11.6 (±5.4)	11.2 (± 5)	ns
BMI	25.8 (±4.0)	27.4 (± 5.2)	ns
Smokers/non‐smokers %	17 (41%)	4 (36%)	ns
Systolic BP mmHg	118.8 (±10.0)	119 (± 9.2)	ns
Diastolic BP mmHg	79 (±7.5)	78 (± 6.8)	ns
eGFR (mL/min)	109 (±16.8)	110 (± 15.4)	ns

*Note*: Mean ± SD. Independent samples *t*‐test. *p < .05.

Abbreviations: BMI, body mass index; DBP, diastolic blood pressure; eGFR, estimated glomerular rate; SBP, systolic blood pressure.

**TABLE 2:** Chemokine levels according to the presence of diabetic polyneuropathy (DPN) in patients with type 1 diabetes (T1D) and in comparison with a control group without diabetes.

	Control group *n* = 19	T1D Without DPN *n* = 41	T1D With DPN *n* = 11
Age (years)	26.5 (**±** 4.5)	26.8 (**±** 3.9)	30.9 (**±** 2.9) *
Female	11 (58%)	15 (29%)	5 (11%)
Male	8 (42%)	26 (50%)	6 (10%)
CCL22 (pg/mL)	1695 (1123–1770)	1269 (713–1687)	1417 (1083–1824)
CXCL8 (pg/mL)	9 (5–24)	6 (3–17)	1417 (1083–1824)
CXCL10 (pg/mL)	599 (458–893)	685 (509–1138)	795 (572–1053)
CCL20 (pg/mL)	34 (5–73)	31 (13–49)	45 (14–51)
CXCL9 (pg/mL)	970 (743–113)	1341 (887–1857)*	2310 (902–4173)**
CXCL11 (pg/mL)	256 (143–455)	272 (137–498)	248 (141–340)
CXCL17 (pg/mL)	10 (4–34)	14 (4–28)	16 (4–36)

*Note*: Age shown as mean ± SD. Chemokines data shown as median (25th–75th percentiles). Mann–Whitney test. *p*‐values versus control subjects, *p < .05, **p < .01.

In total, 52 patients (20 women), 20–35 years of age (mean 27.3 SD ± 4.1), and with a disease duration of 19.5 **±** 5.5 years, were included. For comparison, a control group was included (11 women: mean age 26.5 ± 4.5 years).

All patients were divided into two groups according to the presence of diabetic neuropathy based on neurophysiological examination (including subclinical and clinical neuropathy) patients without DPN n = 41 (15 women) and patients with DPN n = 11 (five women). The frequency of neuropathy was 21% (11/52). Nine of the 11 patients with DPN also had signs of clinical diabetic neuropathy. In addition, four patients from the neuropathy group reported symptoms of sensory neuropathy, such as numbness or pain. In logistic regression analysis for groups with and without diabetes neuropathy in correlation with age and HbA1c, it was found that for every one‐year increase in age, the odds for diabetic neuropathy increased by about 40%, adjusted for HbA1c [OR (95% CI) = 1.399 (1.082, 1.808), *p* = .010].

### 3.2 Chemokines (Figure 1, Table 2)

**FIGURE 1:**
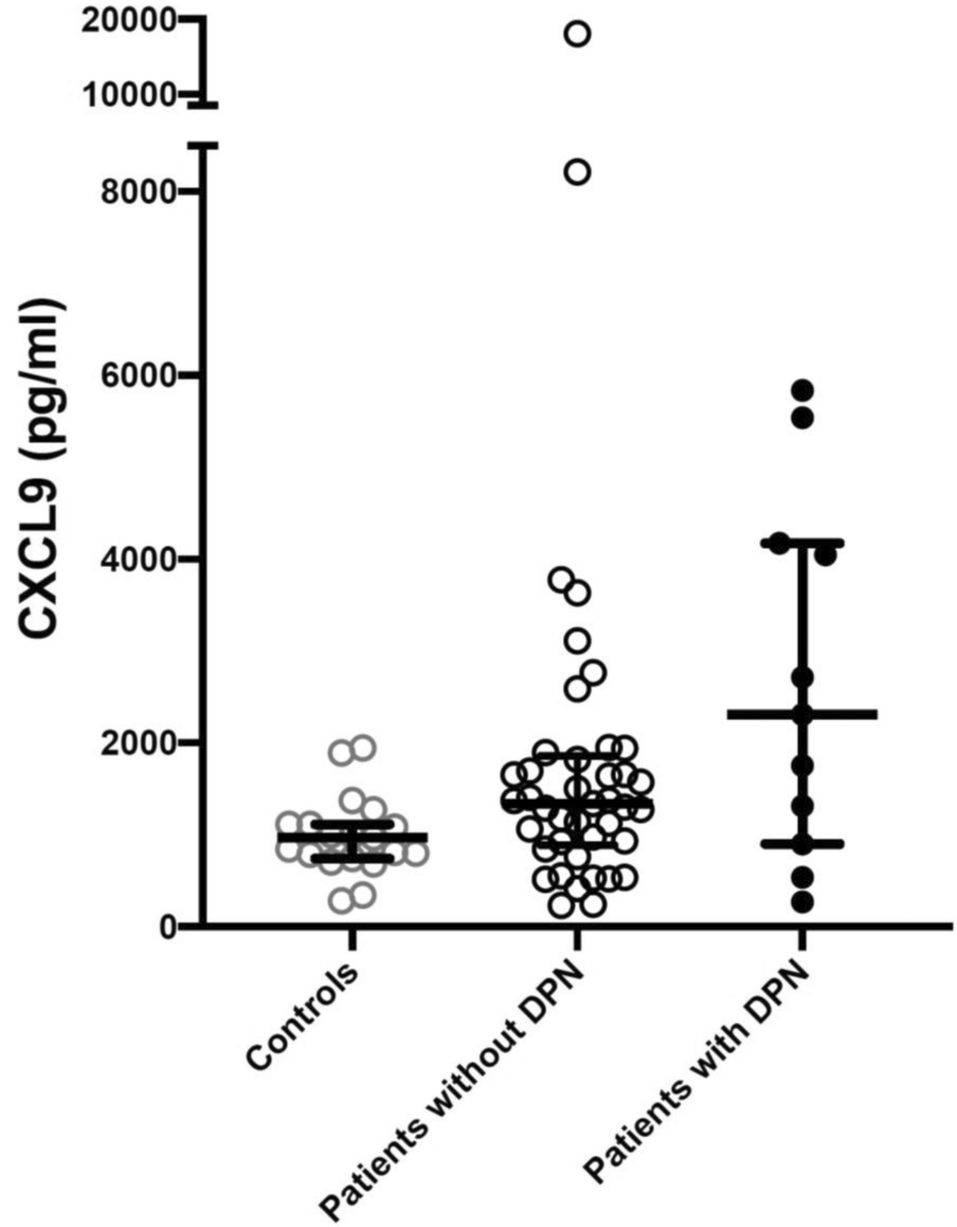
In the Kruskal–Wallis H test, significantly higher CXCL9 levels were found between control subjects and patients with DPN (*p* = .019), while no difference between control subjects and patients without DPN was seen after adjustment for multiple comparisons.

In univariate tests, the Th1‐associated chemokine CXCL9 was significantly higher in patients with T1D than in healthy controls. However, only patients with neuropathy had significantly higher CXCL9 levels compared to controls, *p* = .019, while no difference was found between patients without neuropathy and controls after adjustment for multiple testing. In addition, no significant differences were found between patients with TID with and without neuropathy, or in comparison with healthy controls regarding the other Th1‐associated chemokines (CXCL10, CXCL11), the Th2‐related chemokines (CCL17, CCL22) or the Th17‐associated chemokines CCL20 and CXCL8, after adjustment for multiple testing.

### 3.3 Electroneurography and chemokines (Table 3)

**TABLE 3:** Correlations between chemokine levels and the nerve function tests within the groups of patients without diabetes neuropathy (TID) and with diabetes neuropathy (TID + DPN).

	Peroneus CMV		Peroneus CMAP		Suralis SCV		Suralis SNAP	
	T1D *n* = 41	T1D + DPN *n* = 11	T1D *n* = 41	T1D + DPN *n* = 11	T1D *n* = 41	T1D + DPN *n* = 11	T1D *n* = 41	T1D + DPN *n* = 11
CXCL9	−0.126	−0.336	−0.041	−0.392	0.045	−0.337	0.070	−0.251
CXCL10	0.353	−0.456	0.156	−0.558	0.271	−0.916^**^	0.175	−0.738^**^
CXCL11	**−**0.352	−0.105	0,102	−0.129	−0.129	−0.465	−0.192	−0.082
CXCL17	0.086	−0.588	0.111	−0.392	0.393	−0.311	0.393	−0.506
CCL20	0.190	−0.064	0.348	−0.106	0.211	−0.137	0.277	0.146
CCL22	0.060	−0.118	0.236	0.212	−0.035	0.592	0.041	0.319
CXCL8	−0.005	0.405	−0.042	0.387	0.020	0.00	0.080	0.469

*Note*: Correlations (Spearmans, Rho). *p*‐values are FDR‐adjusted based on the Benjamini–Hochberg method. **P < .01.

Bilateral measurements of both peroneal and median motor nerve conduction velocity (MCV), compound muscle action potential (CMAP) amplitude, sensory nerve action potential (SNAP), and sural and median sensory nerve conduction velocity (SCV) were measured. Significant negative correlations between suralis MCV, suralis SNAP and CXCL10 (rho −0.966, *p* < .001 and rho −0.738, *p* < .001, respectively) were found in T1D patients with DPN after correction and adjustment for multiple testing. No other correlations were found between nerve conduction data and the other chemokines.

### 3.4 Peripheral nerve tests and chemokines (Table 4)

**TABLE 4:** Correlations between chemokine levels and quantitative sensory tests (QSTs) were performed bilaterally according to standardized procedures within the groups of patients without diabetes neuropathy (TID) and with diabetes neuropathy (TID + DPN).

	Vibration perception		Heat tolerance		Cold perception	
	T1D *n* = 41	T1D + DPN *n* = 11	T1D *n* = 41	T1D + DPN *n* = 11	T1D *n* = 41	T1D + DPN *n* = 11
CXCL9	−0.186	0.317	−0.134	0.087	0.142	−0.116
CXCL10	−0.148	0.639^*^	−0.100	0.242	−0.075	0.343
CXCL11	0.124	0.469	−0.133	0.269	0.226	−0.274
CXCL17	−0.165	0.178	−0.197	0.532	0.094	0.204
CCL22	−0.031	−0.446	0.125	0.301	0.258	−0.009
CCL20	−0.404	0.290	−0.444	0.210	−0.227	−0.534
CXCL8	0.024	−0.645	−0.203	−0.037	−0.105	−0.696^*^

*Note*: Correlations (Spearman, Rho). *p*‐values are FDR‐adjusted based on the Benjamini–Hochberg method. *P < .05.

In order to explore possible associations between chemokines and peripheral sensory nerve function, plasma concentrations of chemokines were associated with three tests of sensory perception ability: heat tolerance test, cold perception threshold and vibration perception in which the nerve function is studied in myelinated C, Aδ and Aβ nerve fibres, respectively. For the cold perception, a significant and negative correlation was found for the Th17‐associated chemokine CXCL8 (rho −0.645, *p* = .032) in T1D patients with DPN but not for other chemokines after correction and adjustment for multiple testing. On the contrary, the CXCL10 (Th1) levels were significantly positively correlated to the vibration perception threshold (rho 0.639, *p* = .034) in the T1D + DPN group. No other significant correlations were found between QST and the other chemokines.

### 3.5 Longitudinal follow‐up study

A subgroup of 24 of the cohort of 52 patients was followed up and re‐examined a third time with neurophysiological tests after ten years. Of these, ten patients were still free of clinical and subclinical neuropathy; seven patients had persisting neuropathy, while six new patients had developed neuropathy from the previous follow ten years earlier, giving a frequency of neuropathy of 54% (13/24). No patient had recovered from a prior diagnosis of neuropathy. If the chemokine expression a decade earlier was recalculated in these 24 patients and compared with the former control subjects (n = 19), the same relationship was found that higher levels of CXCL9 levels were expressed in patients with DPN compared with control subjects (*p* = .021), while no difference between patients without DPN and control subjects were seen after adjustment for multiple comparisons.

## 4 DISCUSSION

Recently the role of chemokines in the pathogenesis of diabetic neuropathies has been investigated, mostly in animal studies.^19^ In the present study, the IFN‐ɣ‐induced chemokine CXCL9, which regulates cell trafficking of activated Th1 cells, natural killer cells and monocytes, was elevated for the whole cohort of patients with T1D but significantly higher for the patients with DPN in comparison with the control group. CXCL9 acts through the CXCR3 receptor and has proinflammatory and antiangiogenic effects.^2^, ^11^, ^14^ A previous study also suggested that CXCL9 contributes to the development and progression of other diabetic complications, such as proliferative diabetic retinopathy in patients with type 2 diabetes, which is in line with our findings regarding DPN in T1D.^12^ A recent study from an Iranian cohort of 25 T1D patients supports our results on the key role of CXCL9 in the pathogenesis of DPN.^25^ Despite that, in this last study, the patients had a shorter diabetes duration disease and worse metabolic control. In contrast to previous studies,^26^ elevated serum levels of CXCL10 were not found in patients with T1D compared to healthy controls or between the two groups of T1D individuals. A possible explanation could be the long duration of diabetes disease of 2 decades in our cohort from the onset of diabetes.^2^ This is also the case in another cohort study with patients with T1D and similar diabetes duration.^27^

On the contrary, several negative correlations were found between suralis nerve conduction data and CXCL10 levels, indicating that Th1‐mediated inflammation may be associated with diabetic neuropathy severity. In the same direction, a positive correlation between vibration threshold and CXCL10 levels was found. These findings suggest an association between Th1‐related systemic inflammation and neurotoxicity on the small Aβ fibres. Our findings may align with previous studies showing that hyperglycemia promotes the expression of CXCL10 and CXCL11 on Schwann cells, recruiting CXCR3‐expressing CD8+ lymphocytes and leading to apoptosis of the Schwann cells.^20^

The elevated levels of specific CXCR3 ligands may indicate new therapeutic targets in T1D and, more particularly, in DPN. Chemokine neutralization as part of combination therapy has been evaluated in several clinical trials to prevent further destruction of beta cells.^28^ The presence of negative correlations between the Th17‐induced chemokine CXCL8 in the T1D + DPN group and the peripheral nerve tests is intriguing. It suggests involvement of Th17‐associated immune responses in the pathogenesis of diabetic neuropathy affecting Aδ nerve fibres. Increased levels of CXCL8 chemokines in patients with DPN were correlated with an elevated cold perception threshold, suggesting a more severe DPN. High levels of another Th17‐induced chemokine CCL20 have been reported in studies with patients with T2D.^29^ In addition, Th17 cells may contribute to damage to peripheral neurons through the cytokine IL‐17, which may lead to diabetic neuropathy.^13^ Taken together, therapeutic prospects, which aim to modulate the Th1/Th17 system in relation to disease duration would be exciting to explore in the cases of diabetic complications such as DPN if further deterioration could be inhibited.

There are some limitations to our study. First, although an association was found between CXCL9 and T1D, and several significant correlations between pathological nerve function tests and Th1 and Th17‐induced chemokines were noted, the causality between inflammation factors and DPN cannot be established. It would be interesting due to prospectively designed studies to identify changes in biomarker levels before the onset of DPN and metabolic control. Our patients had a long diabetes duration, and several patients already suffered from DPN. The control subject was well matched due to age, but the proportion of women was more prominent in the control group compared with the patients with type 1 diabetes, which may have affected the results. On the contrary, to our knowledge there is no gender differences in chemokines distribution.

In this novel pilot study that investigated nerve conduction tests and chemokines levels in patients with type 1 diabetes. Another limitation of our study is that the current cohort sample is relatively small but hopefully our results can form the basis of a large prospective study that may give further answers and explanations and facilitate the research on pathogenesis‐derived pharmacotherapy.^30^, ^31^

## 5 CONCLUSIONS

Higher levels of the Th1‐associated chemokine CXCL9 were detected in patients with T1D and DPN after two to three decades of diabetes duration compared to healthy control subjects. Furthermore, Th1 and Th17‐associated chemokines were associated with impaired peripheral sensory nerve function and nerve conduction. Our findings suggest complex immunological pathogenesis behind diabetic neuropathy in type 1 diabetes that may open new therapeutic possibilities.

## AUTHOR CONTRIBUTIONS

**Evangelia Baldimtsi:** Conceptualization (equal); data curation (equal); formal analysis (equal); investigation (equal); methodology (equal); project administration (equal); resources (equal); software (equal); writing – original draft (equal); writing – review and editing (equal). **Nektaria Papadopoulou‐Marketou:** Conceptualization (supporting); data curation (supporting); formal analysis (supporting); methodology (supporting); project administration (supporting); writing – review and editing (supporting). **Maria Jenmalm C:** Conceptualization (equal); data curation (equal); formal analysis (equal); methodology (equal); project administration (equal); supervision (equal); validation (equal); visualization (equal); writing – review and editing (equal). **Jeanette Wahlberg:** Conceptualization (lead); data curation (lead); formal analysis (lead); funding acquisition (lead); investigation (lead); methodology (lead); project administration (lead); resources (lead); software (equal); supervision (lead); validation (lead); visualization (lead); writing – original draft (equal); writing – review and editing (equal).

## FUNDING INFORMATION

Grants from the Swedish state financed this study under the agreement between the Swedish government and the county councils, the ALF agreement, LIO 89939, and by grants from the Region of Östergötland, LIO‐790841 and LIO711061.

## CONFLICT OF INTEREST STATEMENT

The authors declare no conflict of interest.

## APPROVAL OF THE RESEARCH PROTOCOL

The study protocol was approved by the Research Ethics Committee, Linköping, Sweden, No: 2017/190–31, on March 2017.

## INFORMED CONSENT

Written informed consent was obtained by all subjects before participation. The study conforms to the provisions of the Declaration of Helsinki.

## APPROVAL DATE OF THE REGISTRY AND THE REGISTRATION NUMBER OF THIS STUDY

N/A.

## ANIMAL STUDIES

N/A.

## Data Availability

The data generated or analysed during this study are available from the corresponding author upon reasonable request.
